# Monocyte Adhesion, Migration, and Extracellular Matrix Breakdown Are Regulated by Integrin αVβ3 in *Mycobacterium tuberculosis* Infection

**DOI:** 10.4049/jimmunol.1700128

**Published:** 2017-06-23

**Authors:** Sara Brilha, Riccardo Wysoczanski, Ashley M. Whittington, Jon S. Friedland, Joanna C. Porter

**Affiliations:** * Department of Infectious Diseases and Immunity, Imperial College London, London W12 0NN, United Kingdom;; † Centre for Inflammation and Tissue Repair, Respiratory Medicine, University College London, London WC1E 6JF, United Kingdom; and; ‡ Centre for Molecular Medicine, University College London, London WC1E 6JF, United Kingdom

## Abstract

In tuberculosis (TB), the innate inflammatory immune response drives tissue destruction, morbidity, and mortality. Monocytes secrete matrix metalloproteinases (MMPs), which have key roles in local tissue destruction and cavitation. We hypothesized that integrin signaling might regulate monocyte MMP secretion in pulmonary TB during cell adhesion to the extracellular matrix (ECM). Adhesion to type I collagen and fibronectin by *Mycobacterium tuberculosis*–stimulated monocytes increased MMP-1 gene expression by 2.6-fold and 4.3-fold respectively, and secretion by 60% (from 1208.1 ± 186 to 1934.4 ± 135 pg/ml; *p* < 0.0001) and 63% (1970.3 ± 95 pg/ml; *p* < 0.001). MMP-10 secretion increased by 90% with binding to type I collagen and 55% with fibronectin, whereas MMP-7 increased 57% with collagen. The ECM did not affect the secretion of tissue inhibitors of metalloproteinases-1 or -2. Integrin αVβ3 surface expression was specifically upregulated in stimulated monocytes and was further increased after adhesion to type I collagen. Binding of either β3 or αV integrin subunits increased MMP-1/10 secretion in *M. tuberculosis*–stimulated monocytes. In a cohort of TB patients, significantly increased integrin β3 mRNA accumulation in induced sputum was detected, to our knowledge, for the first time, compared with control subjects (*p* < 0.05). Integrin αVβ3 colocalized with areas of increased and functionally active MMP-1 on infected monocytes, and αVβ3 blockade markedly decreased type I collagen breakdown, and impaired both monocyte adhesion and leukocyte migration in a transwell system (*p* < 0.0001). In summary, our data demonstrate that *M. tuberculosis* stimulation upregulates integrin αVβ3 expression on monocytes, which upregulates secretion of MMP-1 and -10 on adhesion to the ECM. This leads to increased monocyte recruitment and collagenase activity, which will drive inflammatory tissue damage.

## Introduction

Tuberculosis (TB) remains an important global health problem with 8.6 million new cases annually, of which at least 480,000 are multidrug resistant (1). Lung cavitation is the hallmark of TB and results from extracellular matrix (ECM) destruction, creating an immuno-privileged site within which mycobacteria can proliferate and spread to new hosts. In addition, tissue damage impairs organ function and results in patient morbidity and mortality. Pulmonary ECM is composed of a network of molecules including type I, III, and IV collagen, fibronectin, laminin, elastin, and proteoglycans. Type I collagen is the primary structural fibril of the lung and is highly resistant to enzymatic degradation. In addition to its biomechanical properties, type I collagen has important roles in cell survival, adhesion, proliferation, and migration (2). Fibronectin is present in lower amounts and has important functions in cell adhesion, growth, and migration (3).

Human monocytes are a key element in the formation of TB granuloma, which is the main cellular host response to infection. Integrins are a family of receptors involved in regulation of immune responses (4), and peripheral blood monocytes express eight integrin heterodimers: α1β1, α3β1, α4β1, α5β1, αXβ2, αMβ2, αLβ2, and αVβ3 (4, 5). These are key in interactions with other cells and with the ECM. Monocyte recruitment in acute inflammation is mediated in part by β2-integrin receptors (6, 7) whereas integrin α4β1 promotes arrest and adhesion to VCAM-1 (8). Engagement of β2-integrins is also involved in downregulation of NF-κB–dependent genes encoding for proinflammatory cytokines via inhibition of TLR signaling (9). Integrin αvβ3 modulates αLβ2 integrin–dependent monocyte adhesion to ICAM-1 (10). *Mycobacterium tuberculosis* infection of macrophages was reported to increase cellular adhesion and decrease surface expression of the phagocytic complement receptors (CR) 3 (integrin αMβ2) and CR4 (integrin αXβ2) (11).

Matrix metalloproteinases (MMPs) are zinc-containing endopeptidases with diverse functions in inflammation and tissue repair. Most MMPs are able to degrade components of the pulmonary ECM, and some are released during the innate inflammatory immune response to *M. tuberculosis* infection. Our group has shown that MMPs are expressed within TB granulomas (12–14) and associated with disease severity (15) and tissue damage (16–18). MMP-1 is the main collagenase responsible for tissue destruction in pulmonary TB (19). In TB patients, including those with TB/HIV coinfection, elevated plasma MMP-1 concentrations were associated with collagen breakdown (20).

In TB, extensive tissue destruction may occur even with a low bacterial load, indicating a role of immune intercellular networks that drive MMP secretion. MMP expression is initially upregulated by *M. tuberculosis–*infected monocytes and macrophages, which then recruit peripheral blood monocytes by secretion of cytokines and chemokine, amplifying this response. MMP-dependent tissue destruction is, therefore, driven by activated uninfected neighboring cells, such as monocytes or respiratory epithelial cells, due to signaling networks within the granuloma (21, 22). MMP-1 is activated from a precursor zymogen (pro–MMP-1), a process which can be promoted by other MMPs, such as the stromelysins MMP-3 and -10 (23, 24). MMP-1 is inhibited by the tissue inhibitors of metalloproteinases (TIMP)-1 and -2 (25–27).

Although integrins are required for adhesion and cellular response to cues from the ECM environment, it is not known whether integrin-dependent adhesion to the lung ECM plays a role in the regulation of MMP gene expression and secretion by human monocytes recruited to the lung in TB. In the current study, we investigated whether contact with ECM components affects the *M. tuberculosis–*associated MMP gene expression and secretion from monocytes and dissected the mechanisms by which integrin signaling regulates MMP secretion in *M. tuberculosis* infection.

## Materials and Methods

### Abs

To study integrin regulation of MMP expression, primary mouse anti-human integrin β1 (clone P4C10), integrin β2 (clone MEM48), integrin β3 (clone B3A), FITC-conjugated anti-human integrin β1, and integrin αV (clone 272-17E6) Abs were used (all from Millipore, Hertfordshire, U.K.). FITC-conjugated goat anti-mouse IgG1 (Sigma-Aldrich, Dorset, U.K.), and Cy5 conjugated goat anti-rabbit (Abcam, Cambridge, U.K.) were used as secondary Abs. Mouse IgG1 and FITC-conjugated mouse IgG1 were the isotype controls (BD Diagnostics, Oxford, U.K.).

### *M. tuberculosis* H37RV culture

*M. tuberculosis* strain H37Rv was cultured in Middlebrook 7H9 medium supplemented with 10% ADC enrichment medium (BD Diagnostics), 0.2% glycerol, and 0.02% Tween 80 (Sigma-Aldrich) with agitation at 10 rpm. For infection experiments, mycobacteria were used at midlogarithmic growth at an OD of 0.60 (Biowave Cell Density Meter; WPA, Cambridge, U.K.).

### Primary monocyte isolation and culture

Ethical approval for obtaining healthy human volunteer blood was provided by the Outer West London Research Ethics Committee and written informed consent was obtained from individuals. PBMCs were isolated by gradient density centrifugation with Ficoll-Paque PLUS (GE Healthcare, Buckinghamshire, U.K.) and CD16 monocytes were purified by negative MACS (MACS monocyte isolation kit II; Miltenyi Biotec, Surrey, U.K.) according to the manufacturer’s instructions. Purity was confirmed by CD64 staining and FACS analysis and was ≥95%. Viability assessed by trypan blue exclusion was ≥98%.

Monocytes were seeded at a density of 2.5 × 10^5^ cells per cm^2^ in RPMI 1640 supplemented with 2 mM glutamine, 10 μg/ml ampicillin, and 10% heat-inactivated FBS. Monocytes were allowed to rest for 1 h before adding 1:5 diluted conditioned medium from *M. tuberculosis*–infected monocytes (CoMtb) or conditioned medium from uninfected monocytes (CoMCont), or infecting with *M. tuberculosis* H37Rv at a multiplicity of infection (MOI) = 1. Monocytes were incubated for 24 h and supernatants were collected for protein concentrations, whereas for investigation of gene expression, monocytes were incubated for 6 h, rinsed with sterile PBS, and lysed with Tri-Reagent.

### CoMtb

Preparation of CoMtb was performed as previously described (28). Briefly, human monocytes cultured in RPMI 1640 (Life Technologies, Paisley, U.K.) medium supplemented with 2 mM glutamine and 10 mg/ml ampicillin, were infected with *M. tuberculosis* strain H37Rv at an MOI of 1, and incubated for 24 h at 37°C and 5% CO_2_. Supernatants were sterile filtered and aliquots stored at −20°C. CoMCont and CoMtb used in each experiment were donor matched.

### Coating of tissue culture plates with ECM components or integrin Abs

Tissue culture plates were precoated with 100 μg/ml native type I collagen from human fibroblasts (VitroCol), 250 μg/ml type IV collagen from human placenta, or 20 μg/ml human plasma fibronectin (all from Advanced BioMatrix, Carlsbad, CA). In brief, for type I collagen, wells were coated at a desired concentration of VitroCol diluted in sterile distilled water and incubated at room temperature for 1 h, rinsed with sterile PBS or HBSS. Type IV collagen was diluted in a 0.25% acetic acid solution and coated plates incubated for 1 h and rinsed with HBSS. Fibronectin was diluted in HBSS, plates incubated for 1 h, and rinsed with sterile distilled water. After incubation, any excess material was aspirated and plates were blocked with sterile 2% heat-denatured BSA, rinsed with PBS or HBSS, and allowed to air dry for at least 45 min.

To find the optimal concentration of ECM ligands, initial titration was performed using the human monocyte cell line THP-1 (Supplemental Fig. 1A–C). Briefly, 96-well plates were coated with increasing concentrations of type I collagen (0, 1, 10, 100, and 200 μg/ml), type IV collagen (0, 10, 100, 250, and 300 μg/ml) and fibronectin (0, 0.2, 2, 20, and 40 μg/ml), and blocked with 2% heat-denatured BSA to prevent unspecific adhesion. Control wells without ECM were only blocked with BSA. THP-1 monocytes were prelabeled with 5 μM calcein, and integrin αMβ2 (Mac-1) was blocked with 10 μg/ml anti-αM Ab (clone LPM19C) to prevent binding to BSA. Then 5 × 10^4^ labeled monocytes were added per well in RPMI 1640 without phenol red, activated with 20 nM PMA, and let to adhere for 1.5 h. A standard curve was generated from serial dilutions of a known concentration of monocytes, and fluorescence measured in a FLUOstar Omega microplate reader (BMG Labtech, Buckinghamshire, U.K.).

To selectively analyze engagement of specific integrins, tissue culture plates were coated with goat anti-mouse IgG H+L polyclonal Ab (Sigma-Aldrich) overnight at 4°C, washed with PBS, and coated for 2 h at room temperature with 20 μg/ml of anti-integrin Abs (Millipore).

### Measurement of MMP and TIMP concentrations

MMP and TIMP concentrations were analyzed by ELISA (Duoset; R&D Systems, Abingdon, U.K.) or by Luminex bead array (Luminex 200; Bio-Rad, Hertfordshire, U.K.) using the Fluorokine MAP kit (R&D Systems) according to the manufacturer’s instructions. Lower limits of sensitivity for the Duoset kits are: 21.2 pg/ml for TIMP-1, 31.2 pg/ml for TIMP-2, 156 pg/ml for MMP-1, and 31.2 pg/ml for MMP-10. In the Fluorokine Luminex kit the lower limits are: 1.1 pg/ml for MMP-1, 12.6 pg/ml for MMP-2, 7.3 pg/ml for MMP-3, 6.6 pg/ml for MMP-7, 13.7 pg/ml for MMP-9, and 3.2 pg/ml for MMP-10. Variation on secreted concentrations of MMPs and TIMP between individual donors is shown in Supplemental datasets.

### RNA extraction and real-time RT-PCR

Total RNA was extracted from monocyte lysates using PureLink RNA Mini Kit (Life Technologies) according to the manufacturer’s instructions. Quantitative real-time RT-PCR was performed using the OneStep RT-PCR Master Mix (Qiagen, Crawley, U.K.) according to the manufacturer’s instruction on a Stratagene Mx3000P platform (SABiosciences, Crawley, U.K.) using 15 μg of RNA per sample. To determine the quantitative change in RNA, standard curves were prepared from a known concentration of the genes of interest and subjected to real-time PCR as above. MMP-1 primers and probes were custom made and supplied by Sigma-Aldrich (forward primer: 5′-AAGATGAAAGGTGGACCAACAATT-3′; reverse primer: 5′-CCAAGAGAATGGCCGAGTTC-3′; probe: 5′-FAM-CAGAGAGTACAACTTACATCGTGTTGCGGCTC-TAMRA-3′), and 18S rRNA primer and probe mix was supplied by Life Technologies.

### RNA extraction and real-time PCR from induced sputum

The study was approved by the University of Cape Town (HREC Ref 516/2011), as previously described (29). Informed consent was obtained in all cases. Later 1 ml of RNA (Qiagen) was added on site to the sputum samples and mucolysis was performed by adding an equal volume of 0.1% DTT (Sigma-Aldrich), and agitating gently at room temperature for 20 min. The mucoid layer was filtered through 100 μm pore-size strainer and centrifuged at 500 × *g* for 10 min. The cell pellet was aspirated and 1.5 ml of cold TRI reagent added before vortexing. Total RNA was extracted using the Purelink RNA Mini Kit (Life Technologies) and real-time RT-PCR was performed using 15 ng RNA and the OneStep RT-PCR Kit (Qiagen) on a Stratagene Mx3000Pro, using integrin β3 and β-actin primers and probes (Life Technologies). Analysis was performed using the Pfaffl comparative cycle threshold (Ct) method, applying the equation: ratio (integrin β3:β-actin mRNA) = E _β3_^ΔCt(calibrator-sample)^/E _β−_^actinΔCt (calibrator-sample)^; E is the real-time PCR efficiency of one cycle in the exponential phase, calculated according to the equation: E = 10[−1/slope]. Samples without a Ct value for integrin β3 after 43 cycles but with a Ct for β-actin were considered negative.

### Flow cytometry

Monocytes were detached with 0.5 M EDTA (Life Technologies), washed with PBS, fixed with cold 4% paraformaldehyde, and blocked with 1% BSA/5% human serum buffer. Cells were incubated for 1 h at 4°C with primary mouse anti-human integrin Ab or mouse isotype IgG1 control and FITC-conjugated anti-mouse secondary Ab. For *M. tuberculosis*–infected monocytes, cells were labeled with the Abs first and fixed for 1 h at 4°C with 4% paraformaldehyde. Flow cytometry was performed on a FACSCalibur cytometer that was calibrated using FACS CaliBRITE beads (BD Biosciences). The baseline forward scatter, side scatter, and FL1H settings were adjusted using an unstained, unstimulated monocyte sample. Mean fluorescence intensities (MFI) were compared after normalization to the isotype control. Data were analyzed using FlowJo vX.0.6 (Tree Star, Ashland, OR). Analysis of integrin αVβ3 in the presence of ECM components in *M. tuberculosis–* or CoMtb-stimulated monocytes was performed by plotting MFI data normalized to respective control cells (with control media or CoMCont) from four different healthy volunteers (*n* = 4).

### Confocal microscopy

Eight-well glass slides were precoated type I collagen and monocytes were seeded and incubated as described. Cells were fixed, blocked, and stained with primary anti-integrin Abs and FITC-conjugated goat anti-mouse secondary Ab. MMP-1 was stained with a mouse anti-human MMP-1 primary Ab (Abcam) and Alexa Fluor 633 conjugated with anti-mouse IgG (Life Technologies) as secondary Ab. Integrin β3 was stained with a mouse anti-human β3 primary Ab and Alexa Fluor 488–conjugated goat anti-mouse IgG secondary Ab. Staining with secondary Ab alone was used as control. DQ collagen–coated slides were used to analyze MMP collagenolytic activity by confocal microscopy. Integrin β3 was blocked using 20 μg/ml functional blocking Ab, and a mouse IgG1 Ab was used as a control. For all experiments DAPI was used as nuclear counterstain. Phalloidin conjugated with Alexa Fluor 633 was used to stain F-actin. Images were scanned on a Leica TCS SP5 confocal microscope equipped with 405 nm diode laser, 488 nm argon laser, 543 nm and 633 nm HeNe lasers, and using the Leica Application Suite 2.6.2 software (Milton Keynes, U.K.). Images were processed using ImageJ software v1.46r (National Institutes of Health, Bethesda, MD).

### Transmigration assay

Briefly, 1 × 10^6^ primary human monocytes were loaded on the apical side of a transwell (5 μm pore) precoated with type I collagen. CoMtb or CoMCont (1:2 dilution) was loaded on the basal side to act as a chemotactic stimulus. Monocytes were let to migrate for 2 h at 37°C, 5% CO_2_. Migrating monocytes were collected from the basal side, centrifuged, and resuspended in 400 μl PBS, and 50 μl of CountBright Absolute Counting Beads (Life technologies) were added to each tube. Then 5.5 × 10^6^ unstimulated monocytes were used to adjust baseline forward scatter and side scatter, and exclude cell debris. The total number of transmigrated monocytes was assessed by comparing the ratio of bead to cell events.

### Adhesion assay

Primary monocytes were stained for 40 min with 10 μM CellTracker Green CMFDA (Life Technologies), washed, and incubated for 1 h with 20 μg/ml mouse anti-human integrin β3, 20 μg/ml mouse IgG1 isotype control Abs, or left untreated. Monocytes were loaded on 96-well plates precoated with type I collagen and incubated with CoMCont or CoMtb for 18 h. Nonadherent cells were removed by rising with PBS and serum-free RPMI 1640 without phenol red was loaded in the wells. Fluorescence was measured in a FLUOstar Omega microplate reader.

### Statistical analysis

Data analysis was performed using GraphPad Prism v5.02 (GraphPad software, La Jolla, CA). Data are presented as mean ± SD of three replicate samples and are representative of at least three independent experiments, unless otherwise stated. Statistical analysis was performed using two-tailed Student unpaired *t* test, or Mann–Whitney or one-way ANOVA with Tukey post hoc analysis were used as appropriate. Differences between variables were considered statistically significant for *p* values <0.05. The *p* values are represented as follows: *p < 0.05, **p < 0.01, ***p < 0.001, ****p < 0.001.

## Results

### Adhesion to the ECM modulates MMP-1 gene expression and secretion by *M. tuberculosis*–infected monocytes

**FIGURE 1.**
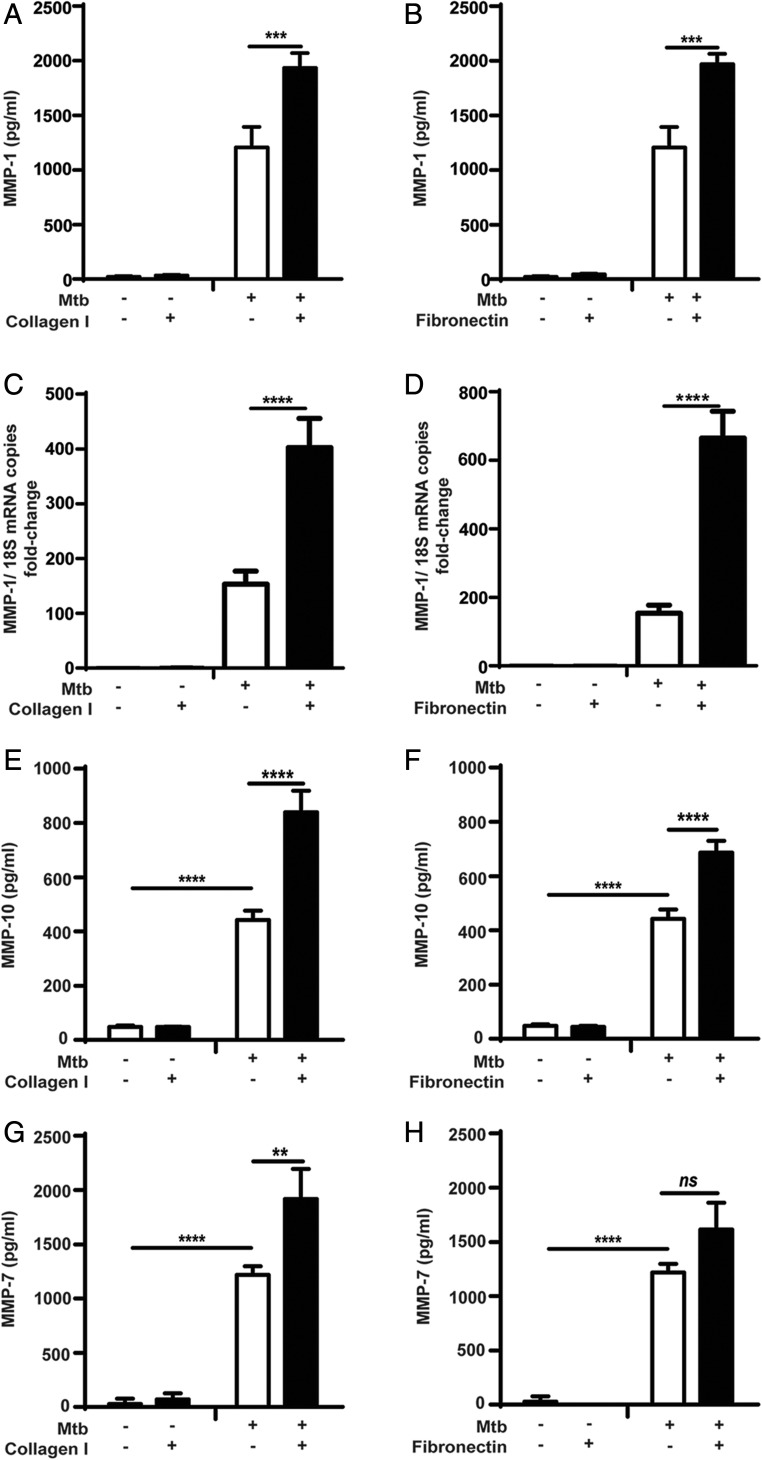
Secretion of MMP-1, -10, and -7 by *M. tuberculosis*–infected primary monocytes is increased by adhesion to type I collagen and fibronectin. Monocytes in the presence or absence of type I collagen, or fibronectin were infected with *M. tuberculosis* (MOI = 1). For secretion analysis, supernatants were collected at 24 h poststimulation, whereas for gene expression cell lysates were collected at 6 h. MMP-1 concentrations were upregulated in *M. tuberculosis*–infected monocytes adherent to (**A**) type I collagen; (**B**) fibronectin, and MMP-1 mRNA accumulation was also upregulated in the presence of (**C**) type I collagen and (**D**) fibronectin. Samples were normalized to 18S rRNA. Secretion of (**E** and **F**) MMP-10, and (**G** and **H**) MMP-7 was also upregulated in the presence of type I collagen and fibronectin, after 24 h of *M. tuberculosis* infection. Graphs show means ± SD and are representative of three independent experiments performed in triplicate. **p < 0.01, ***p < 0.001, ****p < 0.001. ns, not significant.

Because MMP-1 is a key mediator of *M. tuberculosis–*associated immunopathology (19), we investigated the role of components of the ECM on the regulation of MMP-1 gene expression and secretion by primary human monocytes in TB. The optimal coating concentration for each ECM ligand was determined by titration in an adhesion assay using a human monocyte cell line (Supplemental Fig. 1A–C). *M. tuberculosis–*infected monocytes increased MMP-1 secretion after adhesion to collagen or fibronectin by 60% (from 1208.1 ± 186 to 1934.4 ± 135 pg/ml; *p* < 0.0001), and 63% (1970.3 ± 95 pg/ml; *p* < 0.001) respectively (Fig. 1A, 1B). Adhesion to type IV collagen did not alter monocyte MMP-1 secretion compared with cells cultured in the absence of ECM proteins (Supplemental Fig. 1D). At the transcriptional level, MMP-1 mRNA accumulation in *M. tuberculosis*–stimulated human monocytes increased 2.6-fold with adhesion to type I collagen, and 4.3-fold with fibronectin (all *p* < 0.0001; Fig. 1C, 1D). Uninfected monocytes were then stimulated with CoMtb to mimic the cytokine networks between infected and uninfected cells that amplify MMP expression within the granuloma. Stimulation with CoMCont was used as control. MMP-1 secretion by CoMtb-stimulated uninfected monocytes was also increased by adhesion to ECM substrates (Supplemental Fig. 1E, 1F). Again, no significant difference in MMP-1 concentrations was detected with monocyte adhesion to type IV collagen (Supplemental Fig. 1G).

Next, we investigated the effect of the ECM on regulation on other MMPs upregulated by stimulation with *M. tuberculosis*. Infected monocytes secreted considerably higher concentrations of the stromelysin MMP-10, which were increased by an additional 90% after adhesion to type I collagen (from 442.5 ± 35 to 840 ± 78 pg/ml; *p* < 0.0001; Fig. 1E) and by 55% with fibronectin (686.2 ± 44 pg/ml; *p* < 0.0001; Fig. 1F). MMP-3 secreted concentrations were low, and no differences in secretion were seen with adhesion to ECM proteins (Supplemental Fig. 2A, 2B). The elastase MMP-7 was also upregulated following *M. tuberculosis* infection and was further upregulated by 57% with type I collagen (Fig. 1G, 1H). Secretion of MMP-2, a gelatinase, was downregulated in infected monocytes compared with uninfected controls (*p* < 0.0001) but this was not affected by the ECM (Supplemental Fig. 2C, 2D). The secretion of the other major gelatinase, MMP-9, was upregulated by *M. tuberculosis* infection, but this too was not affected by monocyte adhesion to ECM proteins (Supplemental Fig. 2E, 2F).

### ECM and control of TIMP secretion in *M. tuberculosis*– and CoMtb-stimulated monocytes

**FIGURE 2.**
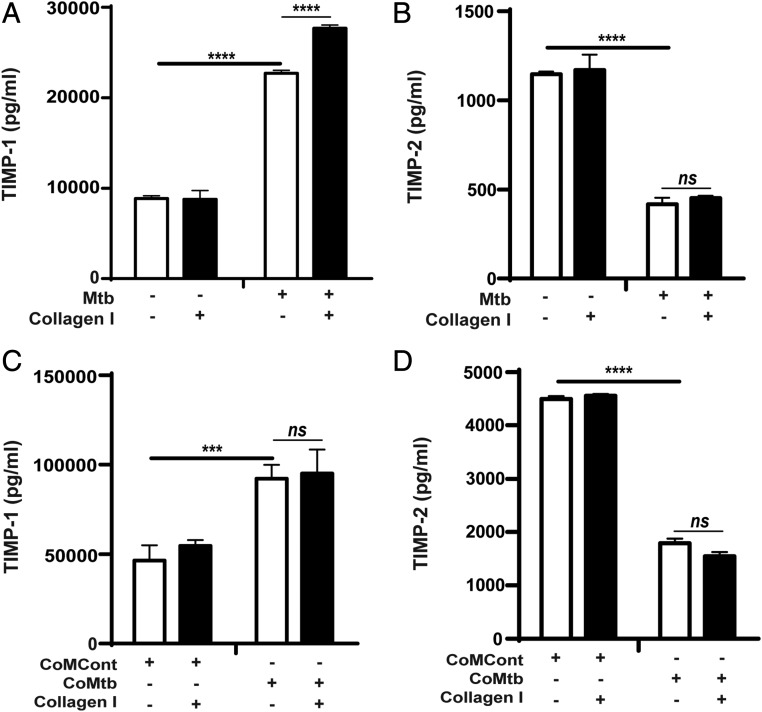
TIMP-1 and -2 secretion by *M. tuberculosis*–infected and CoMtb-stimulated monocytes adherent to type I collagen. Monocytes adherent to type I collagen, fibronectin or in the absence of matrix were either infected with *M. tuberculosis* (MOI = 1) or stimulated with CoMtb (1:5 dilution). Supernatants were collected at 24 h postinfection and analyzed for secreted concentrations of (**A**) TIMP-1 and (**B**) TIMP-2 with *M. tuberculosis*–infected monocytes, and (**C**) TIMP-1 and (**D**) TIMP-2 secretion of CoMtb-stimulated monocytes. CoMCont was used as control of CoMtb. Bars show mean ± SD and data are representative of three independent experiments performed in triplicate. ***p < 0.001, ****p < 0.001. ns, not significant.

TIMPs are critical regulators of MMP activity and MMP:TIMP ratios regulate net tissue destruction, and therefore secreted concentrations of TIMP-1 and -2 were also measured. *M. tuberculosis* infection increased secretion of TIMP-1 by 2.6-fold (from 8.6 to 22.7 ng/ml; *p* < 0.0001; Fig. 2A), whereas TIMP-2 concentrations decreased by ∼64% (Fig. 2B). However, the absolute TIMP-1 concentrations were much greater than the secreted TIMP-2 concentrations. Adhesion to type I collagen increased the TIMP-1 concentrations secreted by *M. tuberculosis*–stimulated monocytes (from 22.71 ± 8.86 to 27.69 ± 8.78 ng/ml; *p* < 0.0001; Fig. 2A). Type I collagen did not affect TIMP-2 (Fig. 2B) and fibronectin had no effect on the secretion of either TIMP-1 or -2 by *M. tuberculosis–*stimulated monocytes (data not shown). CoMtb stimulation similarly increased TIMP-1 and decreased TIMP-2 concentrations and secretion was not modulated by the ECM (Fig. 2C, 2D).

### Integrin expression by human monocytes in TB

**FIGURE 3.**
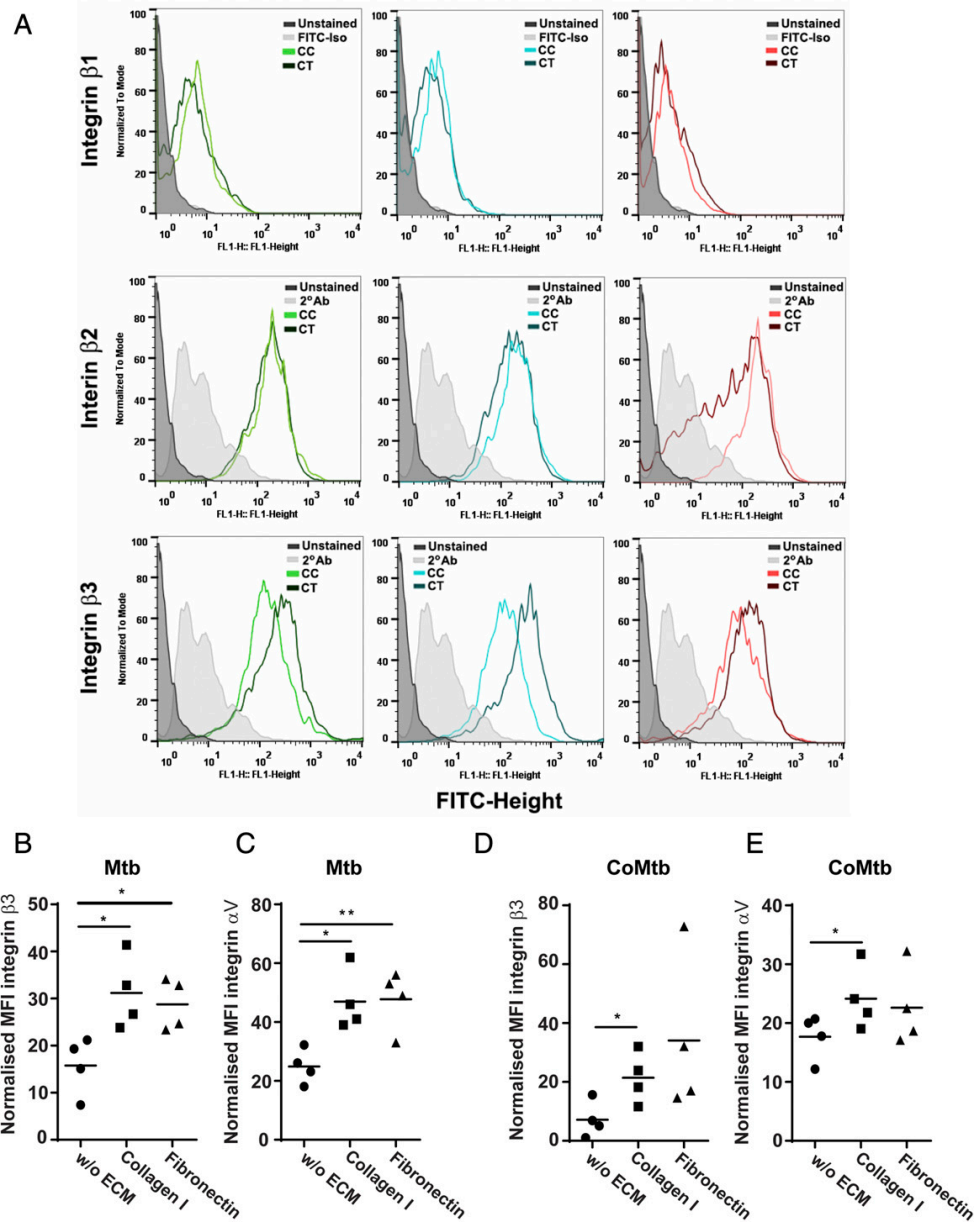
Surface expression of β3 and αV integrin subunits in *M. tuberculosis*–stimulated monocytes adherent to ECM proteins. Monocytes cultured in the presence or absence of type I collagen or fibronectin were stimulated for 24 h with CoMtb (1:5 dilution), and CoMCont as control, or directly infected with *M. tuberculosis* (MOI = 1). Cells were fixed, blocked, and stained with FITC-conjugated anti-integrin β1or primary anti-integrin β2, β3, or αV Abs and secondary FITC-conjugated anti-mouse IgG Ab. Secondary Ab alone or FITC-conjugated IgG1 isotype Ab were used as controls. (**A**) Histograms of integrin subunits β1, β2, and β3 for control and CoMtb-stimulated monocytes show an increase in β3 expression with CoMtb stimulation. MFIs of integrin subunits (**B**) β3 and (**C**) αV in monocytes stimulated with *M. tuberculosis*, and MFIs of (**D**) β3 and (**E**) αV in monocytes stimulated with CoMtb, in the presence or absence of matrix components (*n* = 4). MFIs were normalized to baseline MFIs of respective controls (control media or CoMCont). (B)–(E) show data points and means. *p < 0.05.

Next, the surface expression of β1, β2, and β3 integrin subunits was analyzed to investigate changes in integrin expression in stimulated monocytes compared with controls and in the presence of the ECM (Fig. 3). MFIs for β1 integrin were similar in all conditions, whereas for β2 there was only a small increase with CoMtb stimulation compared with CoMCont. Surface levels of β3 integrin were increased by CoMtb stimulation (Fig. 3A).

Data in which β3 integrin expression in *M. tuberculosis*–stimulated monocytes was normalized by subtracting basal MFIs of the respective control showed that monocyte adhesion to type I collagen and fibronectin increased integrin β3 MFIs by 3-fold (*p* < 0.05) (Fig. 3B). Because the only integrin heterodimer expressed by primary human monocytes containing the β3 subunit is the receptor αVβ3, we next investigated the surface expression of the αV subunit. *M. tuberculosis*–stimulated monocytes adherent to type I collagen had significantly greater integrin αV expression compared with cells cultured in the absence of the ECM (*p* < 0.05; Fig. 3C). Adhesion to fibronectin also significantly upregulated integrin αV expression compared with *M. tuberculosis*–stimulated cells in the absence of the ECM (*p* < 0.01; Fig. 3D). This was also investigated in CoMtb-stimulated monocytes, and we found a similar increase in expression of β3 and αV integrin subunits with adhesion to type I collagen, which was further increased by adhesion to ECM (*p* < 0.05; Fig. 3D, 3E).

### Integrin regulation of MMP secretion in TB

**FIGURE 4.**
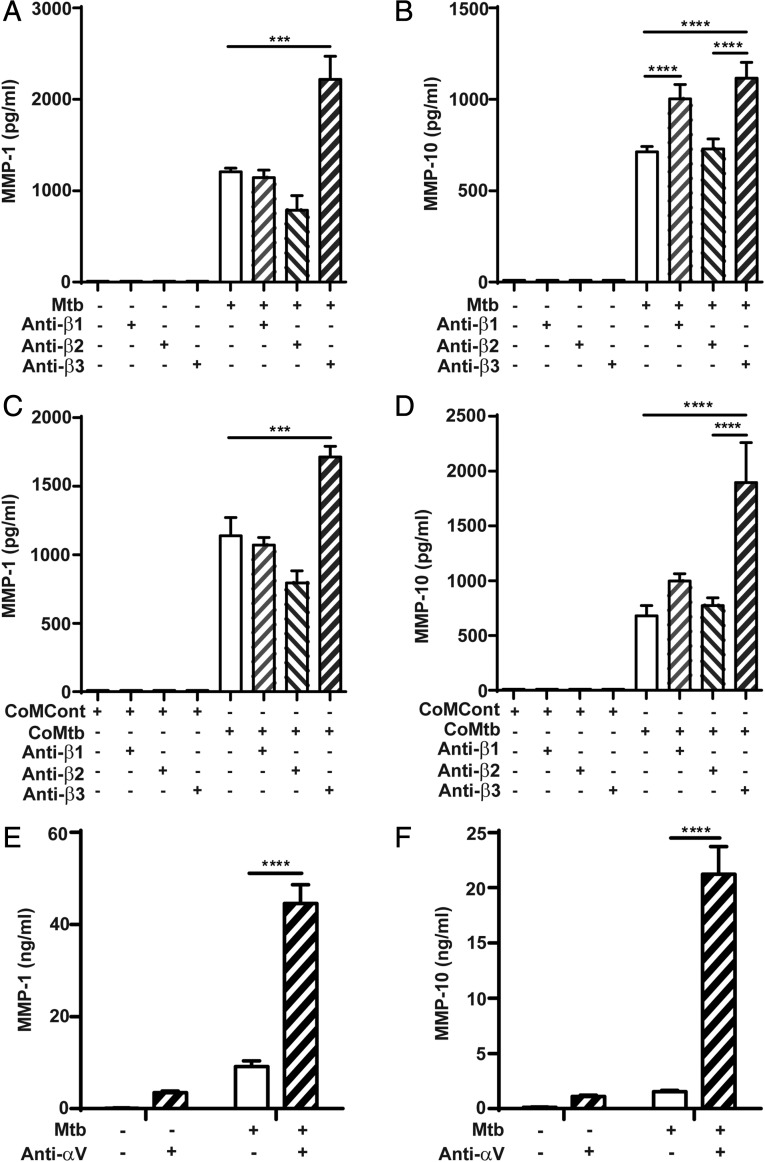
Regulation of MMP-1 and -10 by integrin β and αV subunits in *M. tuberculosis*–infected and CoMtb-stimulated primary monocytes. Plates were precoated with goat anti-mouse Fc region, blocked, and coated with 20 μg/ml either mouse anti-human integrin β1, β2, β3 Abs, or left uncoated and stimulated for 24 h with (**A** and **B**) *M. tuberculosis* or (**C** and **D**) CoMtb. CoMCont was used as control for CoMtb. Supernatants were collected and assayed for MMP-1 and MMP-10. Plates were also coated with 20 μg/ml anti-integrin αV Abs, monocytes infected with *M. tuberculosis* (MOI = 1) and supernatants assayed for (**E**) MMP-1 and (**F**) MMP-10. Graphs show means ± SD and are representative of three independent experiments performed in triplicate. *p < 0.05, ***p < 0.001, ****p < 0.0001.

Next, the regulatory effect of integrin αVβ3 activation on MMP-1 and MMP-10 gene expression and secretion by *M. tuberculosis–* and CoMtb-stimulated monocytes was investigated. Integrin activation was performed by precoating plates with anti-integrin Abs to the ligand binding site to act as ligand mimetic. In *M. tuberculosis*–infected monocytes, activation of the β3 subunit caused an 84% increase in monocyte MMP-1 secretion (from 1207 ± 41 to 2218 ± 253 pg/ml, *p* < 0.001; Fig. 4A), whereas no significant differences were detected after activation of the β1 subunit, and a small decrease in MMP-1 was detected with the β2 subunit (*p* < 0.05). MMP-10 secretion was similarly upregulated by integrin β3 activation following *M. tuberculosis* infection (from 712.8 ± 28.9 to 1115.9 ± 86 pg/ml; Fig. 4B). A significant increase in MMP-10 concentrations was also detected after activation of β1 integrins. Similar data were observed with CoMtb stimulation of human monocytes following activation of the β3 integrin subunit (*p* < 0.001; Fig. 4C, 4D). A small decrease in MMP-1 was also detected with integrin β2 activation (from 1138 ± 133 to 794.5 ± 90 pg/ml). Monocyte MMP-10 secretion was not altered by activation of β1 or β2 integrins in CoMtb-stimulated monocytes. Binding to αV (which pairs with β3 on monocytes) resulted in a 13-fold increase in MMP-1 secretion (*p* < 0.0001; Fig. 4E), and 14.7-fold increase in MMP-10 concentrations (from 1459.73 ± 36.9 to 21543.41 ± 2485.7 pg/ml; *p* < 0.0001; Fig. 4F) following *M. tuberculosis* infection. Binding to αV also increased MMP-1 and -10 secretion from uninfected control monocytes.

### Integrin β3 expression by cells extracted from sputum of TB patients

**FIGURE 5.**
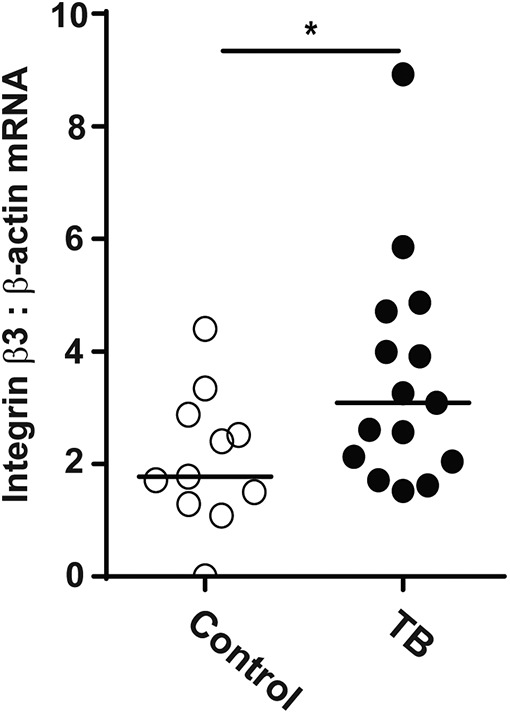
Integrin β3 mRNA is increased in cells from sputum samples of TB patients. Induced sputum samples were collected prospectively from patients with active pulmonary TB (*n* = 15) and control subjects (*n* = 11) in Cape Town. Integrin β3 mRNA accumulation was analyzed by real-time RT-PCR, and β-actin mRNA was used as the control gene. Horizontal lines represent median values. Statistical analysis was performed using a Mann–Whitney *U* test (*p < 0.05).

Because integrin αVβ3 was increased in our cellular model of tuberculosis and its activation was associated with increased MMP-1 and -10 secretion, we next investigated whether this might be happening in TB patients. We investigated induced sputum samples from a cohort of TB (*n* = 15) and control subjects (*n* = 11) from Cape Town, South Africa (Fig. 5). This cohort of TB and control subjects has been previously reported (29). Integrin β3 mRNA accumulation in patients with TB was increased by 78.6% compared with control subjects when normalized to β-actin mRNA (*p* < 0.05).

### Integrin αVβ3, collagen breakdown, and monocyte adhesion and migration in TB

**FIGURE 6.**
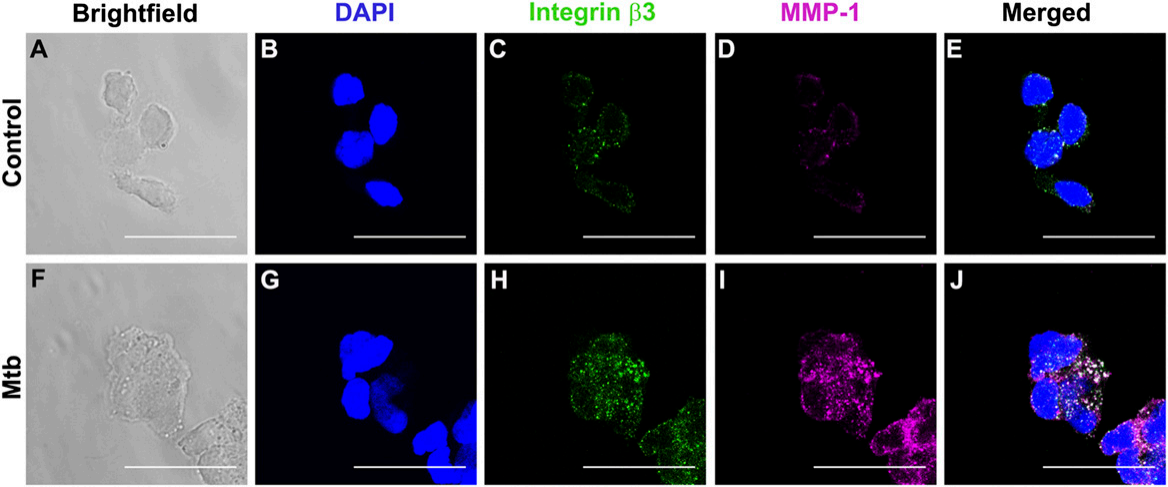
Integrin β3 colocalizes with MMP-1 in *M. tuberculosis* infection. Monocytes seeded in collagen-coated chamber slides were infected with *M. tuberculosis* (MOI = 1). Cells were fixed, blocked, and stained with DAPI for nucleic acids (blue), integrin αVβ3 (green), and MMP-1 (magenta). (**A**) and (**F**) show brightfield and (**B**)–(**D**) show fluorescence of control monocytes and (**G**–**I**) fluorescence of *M. tuberculosis*–infected monocytes. (**E**) and (**J**) are merged fluorescence images. White color corresponds to areas of integrin and MMP colocalization. Scale bar, 25 μm.

Next, confocal microscopy was used to analyze differences in integrin αVβ3 and MMP-1 localization in both uninfected control monocytes (Fig. 6A–E) and *M. tuberculosis*–infected cells (Fig. 6F–J). We observed increased integrin β3 staining with *M. tuberculosis* infection compared with controls (Fig. 6C, 6H). MMP-1 expression was also markedly increased with *M. tuberculosis* infection (Fig. 6D, 6I) and there was colocalization of integrin αVβ3 with MMP-1 in infected monocytes (white staining, Fig. 6J).

**FIGURE 7.**
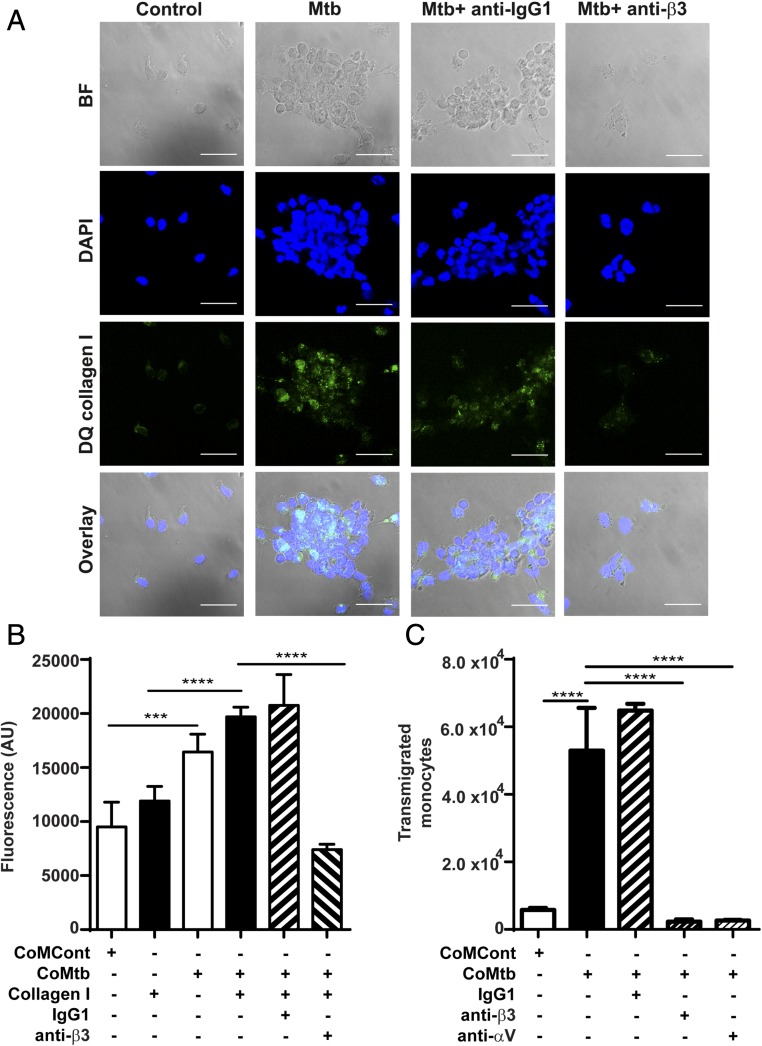
Integrin αVβ3 upregulates type I collagen breakdown and increased monocyte adhesion and transmigration in *M. tuberculosis* infection. Slides were precoated with DQ type I collagen in which FITC is quenched and fluorescence is released only in areas of collagen degradation. Monocytes were preincubated with or without a function blocking anti-integrin β3 Ab or anti-IgG1 isotype control, followed by *M. tuberculosis* infection (MOI = 1). (**A**) Panels show brightfield, DAPI nuclear counterstain (blue), collagen degradation (green), and merged images. Scale bar, 25 μm. (**B**) CoMtb stimulation increased monocyte adhesion compared with CoMCont, which was blocked by inhibition of integrin β3. Next 1 × 10^5^ monocytes per well were prestained with CellTracker Green CMFDA dye were stimulated with CoMtb or CoMCont. Integrin β3–mediated adhesion was inhibited with anti-integrin β3 Ab. Control monocytes preincubated with IgG1 isotype Abs. (**C**) Monocyte migration is increased with CoMtb stimulation, which is blocked with inhibition of integrin αV or β3. Transwells were precoated with type I collagen, and CoMtb or CoMCont was added to the basal side in a 1:2 dilution. Integrins were blocked with anti-integrin αV and β3 Abs, or an IgG1 isotype control Ab. Bars show mean ± SD. Data are representative of three independent experiments performed in triplicate. ***p < 0.001, ****p < 0.0001.

To investigate the functional consequences of integrin αVβ3 binding, which resulted in increased MMP-1 secretion, DQ collagen–coated slides were used to analyze type I collagen breakdown. Increased DQ collagen fluorescence, which corresponds to areas of collagen breakdown, was markedly increased with *M. tuberculosis* infection, compared with uninfected controls, but when integrin β3 was inhibited by 20 μg/ml anti-integrin β3 Abs, collagen breakdown decreased, as well as the number of cells adherent to the collagen-coated slide, which further implicates integrin αVβ3 on regulation of MMP-1 activity in TB (Fig. 7A). To analyze integrin αVβ3–mediated monocyte adhesion to type I collagen in TB, monocytes labeled with CellTracker Green dye were seeded in either type I collagen-coated or uncoated wells prior CoMtb or CoMCont stimulation. Monocyte adhesion was significantly increased with CoMtb stimulation (*p* < 0.001, Fig. 7B) and was higher in the presence of type I collagen. Inhibition of integrin β3 activity with blocking Abs markedly decreased cell adhesion with CoMtb stimulation. Next, transwell assays were performed to analyze integrin αVβ3 involvement in monocyte migration in TB. With CoMtb stimulation, the number of transmigrated monocytes was 2.2-fold higher than in CoMCont controls (*p* < 0.0001; Fig. 7C). However, blockade of either β3 or αV integrin subunits led to a dramatic 96 and 92% decrease in monocyte transmigration respectively (both *p* < 0.0001), demonstrating a key role in monocyte migration. No change in monocyte migration was seen with blockade of β1 integrins (Supplemental Fig. 3).

## Discussion

In this study, to our knowledge we demonstrate for the first time in *M. tuberculosis* infection that human monocyte adhesion to type I collagen and fibronectin, which are key in the lung matrix, significantly increase MMP-1, -7, and -10 gene expression and secretion, and that this is regulated via activation of integrin αVβ3 on monocytes and in TB patients. In contrast, the gelatinases MMP-2 and -9, which are also secreted in response to TB, were not affected by monocyte binding to ECM.

We show that type I collagen and fibronectin increase MMP-1 gene expression and secretion by monocytes after direct *M. tuberculosis* infection as well as in response to CoMtb, which mimics in vivo *M. tuberculosis*–dependent signaling networks between infected and uninfected cells. However, type IV collagen, found in basement membranes, did not induce a similar effect, which indicates that this response is ligand specific. Monocyte-derived MMP-10 was also upregulated by adhesion to type I collagen and fibronectin. Although stromelysins are not able to degrade type I collagen, they are functionally important in TB because they may activate collagenases such as MMP-1. MMP-3 is secreted at low concentrations by *M. tuberculosis*–infected monocytes, and is therefore less critical than MMP-10. In contrast, gelatinase secretion was not affected by binding to the ECM. Type I collagen had minimal effects on TIMP-1 secretion, which is increased by *M. tuberculosis* stimulation, or on TIMP-2 secretion, which was decreased by TB.

In dissecting the mechanisms by which the lung matrix increased MMP-1 and -10 secretion, we observed that *M. tuberculosis* infection was associated with increased surface expression of the integrin β3 subunit. There were no significant differences in expression of the integrin subunits β1 and β2 in infected monocytes, although they do have other immunological functions in TB (30, 31). CoMtb stimulation of monocytes increased αVβ3 surface expression, which rose further in the presence of type I collagen. Activation of αVβ3 integrin resulted in increased expression of MMP-1 and -10. This is consistent with a murine study showing that overexpression of αvβ3 in breast cancer cells increased the formation of osteolytic lesions, indicating a role for this integrin in driving tissue damage (32). Although not directly involved in adhesion to native type I collagen in healthy tissues, in inflammation RGD-binding motifs within the collagen fibrils become exposed, allowing adhesion of αVβ3 (33, 34), which provides a positive feedback loop for MMP-1 and -10 expression. This is consistent with our observation that inhibition of integrin αVβ3 in *M. tuberculosis*–stimulated monocytes led to decreased type I collagen breakdown and decreased monocyte adhesion. These findings are potentially clinically important in TB patients because to our knowledge we have demonstrated for the first time that the β3 subunit was upregulated in induced sputum of patients with active TB compared with control subjects.

Importantly, integrin αVβ3 was specifically required for monocyte migration and blockade of either αV or β3 subunits dramatically impaired migration in a transwell system. In the zebrafish model of TB, macrophage migration was central to the dissemination of infection to new sites in an MMP-9–dependent manner (35), and excessive leukocyte migration may lead to adverse effects in TB, including tissue damage (28). Confocal microscopy revealed integrin αVβ3 microclustering and areas of colocalization between integrin αVβ3 and MMP-1, which tended to localize at monocyte protrusions, consistent with trailing uropods of migrating monocyte or areas of monocyte-monocyte adhesion.

Taken together the present work shows that, in *M. tuberculosis–*infected monocytes, increased integrin αVβ3 expression, which is also found in TB patients, promotes increased monocyte adhesion to ECM, and upregulates MMP-1 and -10 secretion, which in turn will drive ECM breakdown. The ECM thereby regulates enzymes required for the influx of inflammatory monocytes, which are also involved in driving tissue damage leading to morbidity and mortality in TB. Leukocyte-ECM interactions are potential targets to develop host-directed therapy aimed at reducing the inflammation and matrix degradation that characterize infection with *M. tuberculosis*.

## Supplementary Material

Data Supplement
