# Supplementary material for: Monocyte Adhesion, Migration, and Extracellular Matrix Breakdown Are Regulated by Integrin αVβ3 in Mycobacterium tuberculosis Infection
Source: J Immunol. 2017 Jun 23;199(3):982–91. doi: 10.4049/jimmunol.1700128 (PMC5523580; doi:10.4049/jimmunol.1700128)
Supplement: Data Supplement [file JI_1700128.zip › JI_1700128_Supplemental_Material_1.pdf]

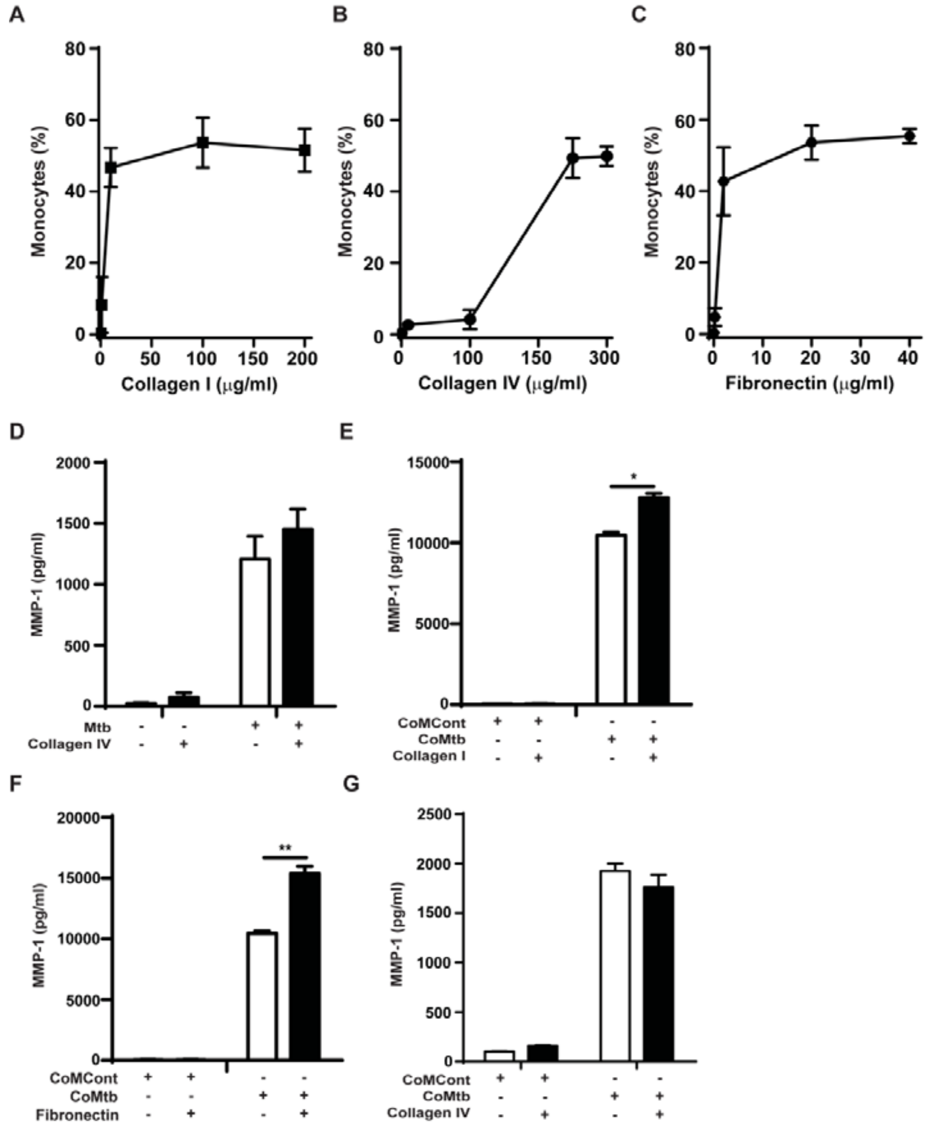

Figure S1. MMP-1 secretion and gene expression by Mtb-infected or CoMtb-stimulated primary monocytes is increased by adhesion to ECM proteins.

(**A-D**) Concentrations of (**A**) type I collagen; (**B**) type IV collagen, and (**C**) fibronectin were titrated using human monocyte cell line THP-1. 96-well plates were coated with increasing concentrations of ECM components and blocked with 2% BSA to prevent unspecific adhesion. Wells without matrix were only blocked with BSA. THP-1 monocytes were pre-labelled with 5μM Calcein and Mac-1 (integrin αMβ3) was blocked with 10μg/ml anti-αM antibody (clone LPM19C) to prevent binding to BSA. $5X10^4$ labelled monocytes were added per well in RPMI with 20nM PMA and without phenol red and let to adhere for 1.5h. A

standard curve was generated from serial dilutions of a known concentration of monocytes, and fluorescence measured in a microplate reader. Figure show means and (n=2) ±s.d. of two independent experiments, each performed in triplicate. **D-G**) MMP-1 secreted concentrations by Mtb-stimulated monocytes. (**D**) MMP-1 concentration in Mtb-stimulated monocytes in the presence of type IV collagen. MMP-1 secretion by CoMtb-stimulated monocytes in the presence of: (**E**) type I collagen; (**F**); fibronectin; and (**G**) type IV collagen. Control media and CoMCont were used as respective controls. Figures show means ±s.d. and is representative of 3 independent experiments performed in triplicate. $*p<0.05$; $**p<0.01$.

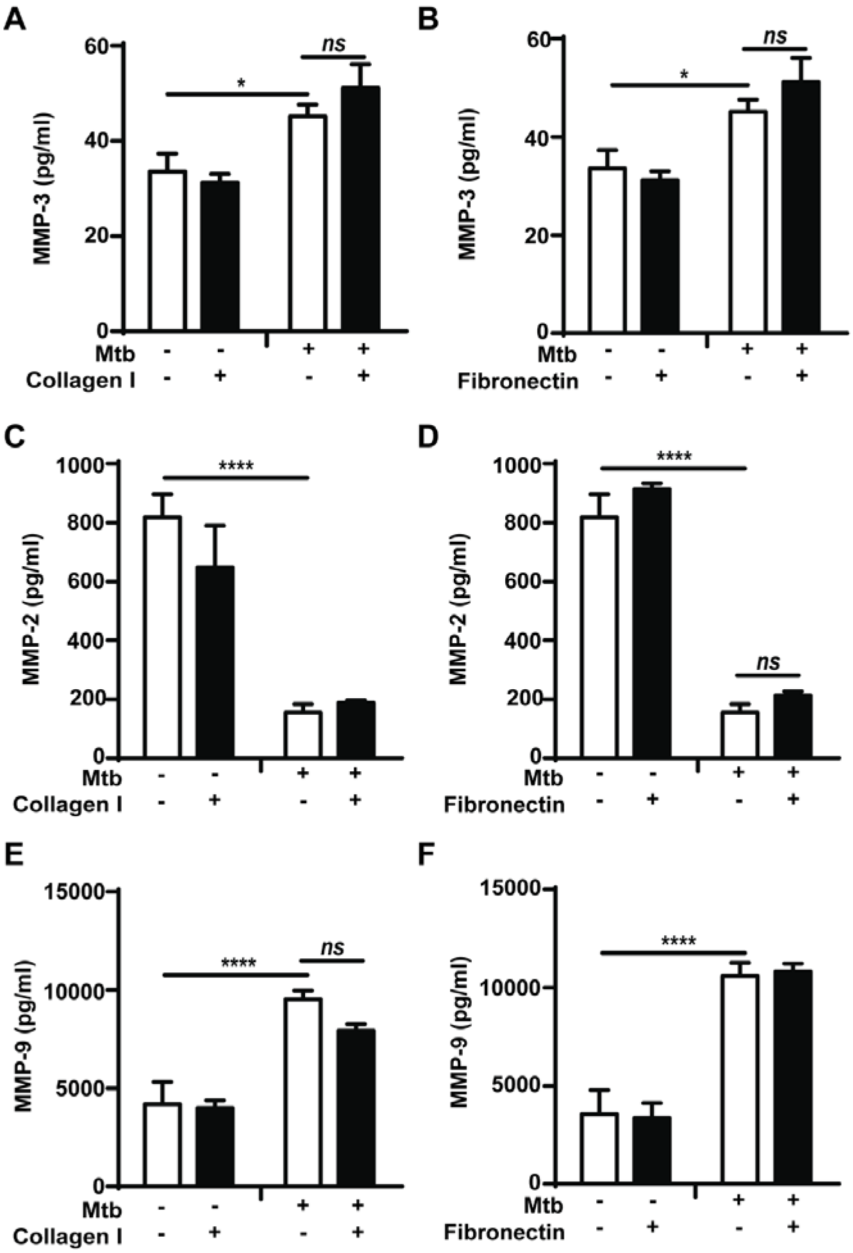

Figure S2. Secretion of MMP-3 and gelatinases MMP-2 and -9 by Mtb-infected monocytes adherent to type I collagen and fibronectin.

Monocytes in the presence or absence of type I collagen and fibronectin or laminin were infected with Mtb (MOI=1). Supernatants were collected at 24h post-infection and analyzed for secreted concentrations of: (**A, B**) MMP-3, (**C, D**) MMP-2, and (**E, F**) MMP-9. Figures show means ±s.d and are representative of 3 independent experiments performed in triplicate. *$p<0.05$; ****$p<0.001$; ns- not significant.

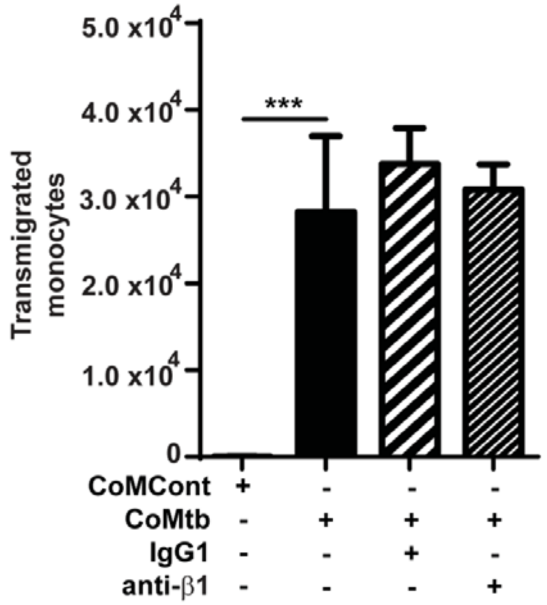

Figure S3. Inhibition of β1 integrins does not affect monocyte migration in Mtb infection.

Monocytes were pre-incubated with or without a function blocking anti-integrin β1 antibody or anti-IgG1 isotype control antibody prior to loading on type I collagen coated tans-wells. CoMtb or CoMCont was added to the basal side in a 1:2 dilution as a chemotactic stimulus. Monocyte migration is increased with CoMtb stimulation, and it is not affected by blockade of β1. Bars show mean ±s.d. Figures are representative of 2 independent experiments performed in triplicate. ***$p<0.001$.

Fig. 1A, B

		No ECM			Collagen I			Fibronectin			Collagen IV		
Donor 1	Cont	18.0	25.4	25.4	37.6	32.8	25.4	47.6	47.6	40.2	114.8	64.8	45.2
	Mtb	1373.5	1244.6	1006.0	2085.8	1890.4	1827.0	1983.3	2057.9	1869.6	1257.1	1523.6	1569.6
Donor 2	Cont	33.4	42.0	42.0	85.2	76.6		67.9	67.9	67.9			
	Mtb	102.7	120.2	137.9	164.7	200.9	173.7	210.1	182.7	210.1			
Donor 3	Cont	34.9	36.4		56.7	36.4		47.1	48.7	53.5			
	Mtb	1649.0	1401.0		1829.6	2022.4	1964.9	2217.9	2369.4	2093.7			

Fig. 1E, F

		No ECM			Collagen I			Fibronectin		
Donor 1	Cont	54.5	44.2	45.5	48.7	49.3	45.5	48.0	43.0	41.1
	Mtb	418.5	426.5	482.5	821.1	926.4	773.6	702.9	636.2	719.6
Donor 2	Cont	51.3	49.3	53.2	48.0	48.7	46.1	61.0	67.6	46.1
	Mtb	2526.3	2595.0	2544.7	4206.6	4370.5	4245.9	3836.7	4222.1	4379.8
Donor 3	Cont	24.0	54.8	9.5	24.0	9.5	39.2	24.0	29.5	39.2
	Mtb	2414.3	2059.4	2504.3	3053.9	2617.4	2891.9	3053.9	2504.3	3059.4

Fig. 1 G,H

		No ECM			Collagen I			Fibronectin		
Donor 1	Cont	85.5	85.5	0.0	50.3	30.9	134.0	0.0	0.0	0.0
	Mtb	1307.3	1154.3	1196.8	1968.2	2167.5	1620.3	1884.6	1395.7	1564.6
Donor 2	Cont	0.0	0.0	0.0	0.0	85.5	0.0	0.0	0.0	0.0
	Mtb	118.2	149.4	164.5	285.2	305.4	384.3	208.5	345.2	332.1
Donor 3	Cont	103.8	100.3	103.1	98.1	112.0	103.8	80.1	84.8	76.8
	Mtb	410.4	364.8	352.3	550.9	619.2	436.9	373.6	380.3	408.3

Fig S1D

		w/o ECM			Collagen IV		
Donor 2	Cont	21.1	20.9	17.3	26.0	18.9	23.0
	Mtb	1939.8	1889.9		1809.9	1993.7	
Donor 3	Cont	90.0	96.9	90.0	111.0	98.7	112.7
	Mtb	1799.7	1431.3	1445.1	1858.7	1665.8	2092.2

Fig S1E, F, G

		w/o ECM			Collagen I			Fibronectin			Collagen IV		
Donor 1	CoMCont	40.6	44.9	60.6	58.8	60.6	53.1	54.5	57.9	39.7			
	CoMtb	10545.6	10587.6	10219.1	13079.0	12681.7	12549.4	14750.4	15411.3	15968.6			
Donor 2	CoMCont	93.9	120.2	137.9	247.2	219.3	304.1	155.7	219.3	266.0	146.8	146.8	137.9
	CoMtb	2126.1	2314.8	2314.9	3072.2	3008.6	2571.9	2977.3	3406.0	3552.8	2036.7	1971.7	2242.5
		w/o ECM		Collagen I		w/o ECM		Fibronectin			Collagen IV		
Donor 3	CoMCont	195.7	220.6	142.1	179.8	89.3	75.3	63.1	72.3	79.4	70.7	82.8	66.7
	CoMtb	1753.9	1445.3	2749.5	2548.6	1788.6	1833.2	2180.1	2196.2	3547.2	1616.3	1684.7	
		w/o ECM			Collagen IV								
Donor 4	CoMCont		104.7	104.7	148.5	163.8	163.8						
	CoMtb	1938.3	1847.4	1993.9	1672.7	1907.8	1705.4						

Fig. S2A, B

		w/o ECM			Collagen I			Fibronectin		
Donor 1	Cont	29.4	36.5	34.9	33.3	30.2	30.2	30.2	28.6	28.6
	Mtb	43.0	44.6	47.9	51.2	56.1	46.2	52.0	51.2	44.6
Donor 2	Cont	14.9	21.9	21.9	14.9	21.9	21.9	21.9	14.9	21.9
	Mtb	43.0	50.0	43.0	36.0	43.0	43.0	36.0	36.0	39.5
Donor 3	Cont	0.0	0.0	0.0	0.0	0.0	0.0	0.0	0.0	0.0
	Mtb	224.3	189.0	208.5	180.3	189.5		172.7	153.2	

Fig. S2C, D

		w/o ECM			Collagen I			Fibronectin		
Donor 1	Cont	753.4	798.5	904.7	613.8	524.8	803.7	891.4	918.0	930.5
	Mtb	134.4	187.1	145.0	197.7	187.1	179.2	197.7	218.8	224.1
Donor 2	Cont	529.1	529.1	529.1	709.3	600.9	893.4	573.8	663.8	573.8
	Mtb	186.4	227.4	227.4	186.4	186.4	227.4	146.1	166.1	227.4
Donor 3	Cont	466.1	510.4	385.5	337.5	348.2	379.2	464.2	479.3	428.1
	Mtb	207.7	237.1	253.1	189.9	191.6	181.2	206.1	210.9	

Fig. S2 E, F

		w/o ECM			Collagen I			Fibronectin		
Donor 1	Cont	4282.2	3003.1	5256.0	3819.9	3731.5	4429.5		2851.4	3908.0
	Mtb	9403.6	10001.5	9152.3	7926.1	7607.7	8262.2	10359.9	11139.1	10951.8
Donor 2	Cont	583.7	233.7	211.8	834.4	170.9	270.7	407.2	381.4	408.2
	Mtb	1660.6	1769.7	1613.7	1362.9	1492.2	1195.6	1932.5	1044.6	1263.6
Donor 3	Cont		1482.5	1474.0	1540.3	1666.6	1341.1		1278.2	1329.0
	Mtb	5268.3	5254.5	4944.0	5224.5	5221.0	4946.3	4100.1	4728.9	4183.8

Fig. 2A

		No ECM			Collagen I		
Donor 1	Cont	8754.6	9196.7	8615.5	17875.3	15016.9	15454.6
	Mtb	22955.3	22848.5	22316.2	27836.6	27949.6	27273.7
Donor 2	Cont	25448.2	28995.9	30114.1	27359.8	31120.6	36923.2
	Mtb	23781.3	23495.0	21669.2	38595.8	36124.6	36369.9
Donor 3	Cont	24476.7	23351.4	20371.9	25577.8	29165.4	19877.6
	Mtb	36113.5	45136.0	36302.2	34645.0	29037.2	45355.6

Fig. 2B

		No ECM			Collagen I		
Donor 1	Cont	1131.7	1160.2	1150.7	1974.3	1790.4	1745.1
	Mtb	413.3	384.1	456.8	466.4	442.3	447.1
Donor 2	Cont	3255.4	4112.9	4057.4	4801.9	3984.2	3993.3
	Mtb	942.4	878.2	1051.9	878.4	871.8	1000.3
Donor 3	Cont	45911.6	45179.6	33321.9	45180.0	29700.3	58023.6
	Mtb	16311.2	11098.4	17726.0	10604.4	13516.5	27862.5

Fig. 2C

		No ECM			Collagen I		
Donor 1	CoMCont	47521.1	37173.5	54373.9	53062.4	58366.4	52589.2
	CoMtb	98222.7	101035.0	87777.0	94539.9	108602.4	81989.8
Donor 2	CoMCont	28234.6	28000.9	32551.7	25794.5	30824.0	28644.1
	CoMtb	39596.3	40351.3	43284.6	40540.7	41491.6	42898.4
Donor 3	CoMCont	5521.2	6204.7	5241.8	6512.8	4359.0	4330.7
	CoMtb	7501.8	7936.7	8828.3	8350.6	9285.1	6903.3

Fig. 2D

		No ECM			Collagen I		
Donor 1	CoMCont	4446.5	4550.8	4483.5	4550.8	4513.3	4588.4
	CoMtb	1750.1	1725.0	1892.1	1640.2	1497.4	1502.3
Donor 2	CoMCont	1237.3	1195.3	1056.7	719.4	986.3	1048.4
	CoMtb	554.9	522.3	541.9	515.8	548.4	515.8
Donor 3	CoMCont	1129.6	1168.5	1071.3	1116.6	1214.2	1325.6
	CoMtb	641.5	737.4	737.4	769.4	782.2	699.1

Datasets of MMP-1, -2, -3, -7, -9, and -10 and TIMP-1/-2 secretion for individual donors.

Datasets that show the variation on MMP and TIMP secretion levels between different donors. A representative donor was used to illustrate the changes on MMP/TIMP secretion by Mtb or CoMtb-stimulated monocytes on figures 1, 2, S1, and S2.
